# Enhanced Liver Targeting of Camptothecin via Conjugation with Deoxycholic Acid

**DOI:** 10.3390/molecules24061179

**Published:** 2019-03-26

**Authors:** Linxia Xiao, Endian Yu, Hanlin Yue, Qingyong Li

**Affiliations:** 1 Collaborative Innovation Center of Yangtze River Region Green Pharmaceuticals, Zhejiang University of Technology, Hangzhou 310014, China; xiaolinxiaxlx@126.com; 2 College of Pharmaceutical Science, Zhejiang University of Technology, Hangzhou 310014, China; yu19960216@126.com (E.Y.); yhl333666@126.com (H.Y.)

**Keywords:** camptothecin, bile acid, liver targeting, antitumor activity, uptake, biodistribution

## Abstract

Camptothecin (CPT) shows potent anticancer activity through inhibition of topoisomerase I. However, its water insolubility and severe toxicity limit its clinical application. Coupling with bile acid moieties is a promising method for liver-targeted drug delivery, which takes advantage of the bile acid receptors on hepatocytes. In this study, we evaluated the potential liver targeting and stability of a deoxycholic acid-CPT conjugate (**G2**). The competitive inhibition of antitumor activity experiment based on bile acid transporters was performed using the MTT method. The effects of deoxycholic acid on uptake of **G2** and CPT were assessed in 2D and 3D HepG2 cell models. The stability of **G2** and CPT was evaluated in vitro (in simulated gastric fluid, simulated intestinal fluid, and fresh rat plasma). Finally, biodistribution of **G2** and CPT was investigated in Kunming mice following oral administration. The results showed that deoxycholic acid pretreatment could significantly reduce the antitumor activity and cellular uptake of **G2** in HepG2 cells, but had no distinct effects on CPT. Meanwhile, **G2** exhibited better stability compared with CPT. More importantly, biodistribution study in mice demonstrated that the liver targeting index of **G2** increased 1.67-fold than that of CPT. Overall, the study suggests that conjugation with deoxycholic acid is a feasible method to achieve liver targeting delivery of CPT.

## 1. Introduction

**Figure 1:**
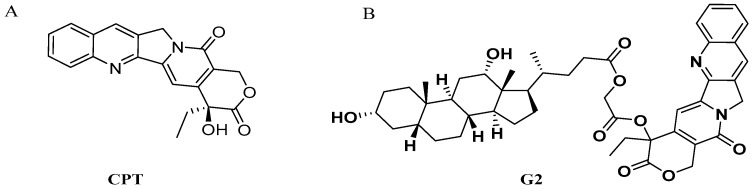
Chemical structures of CPT (**A**) and **G2** (**B**).

CPT (Figure 1A), a natural monoterpene-quinoline alkaloid first isolated from extracts of the Chinese tree *Camptotheca acuminata*, exhibits anticancer activity through inhibition of topoisomerase I [1,2,3,4]. As a potent inhibitor of DNA topoisomerase I, CPT shows high activity against a broad spectrum of cancers, including breast, small-cell lung, ovarian, pancreas cancer, particularly hepatic and colon cancer [5,6,7,8]. Unfortunately, the clinical application of CPT has been hindered by its water insolubility, severe toxicity to normal tissues and poor stability of the lactone ring [9,10,11]. Considerable modifications have been made to overcome these disadvantages. Among the synthesized analogues, irinotecan and topotecan have been approved for clinical therapy of certain cancers, including colon cancer, ovarian cancer, lung cancer and so on [12]. However, toxicities to normal tissues of both drugs are severe because of the non-targeted effects, which limits their utilization [13]. Therefore, the targeted delivery of CPT may lower its accumulation in other tissues and reduce its side effects. 

The prodrug strategy, applying bile acids as the modifying moieties to target hepatocyte transporters and thus obtain hepatic-targeting delivery agents, has been suggested in previous studies [14,15,16,17]. This prodrug approach takes advantage of the highly specific interaction of bile acid ligands with Na^+^-dependent taurocholate co-transporting polypeptide (NTCP) receptors which are specifically and abundantly expressed in hepatocytes [18,19,20]. Furthermore, as a part of the enterohepatic circulation, happening 6–15 times per day, bile acids possess potent transport capacity [21]. Hence, by coupling bile acid moieties to CPT, the uptake of drug-loaded systems into hepatocytes could be enhanced with a high degree of selectivity and the toxicities of the obtained conjugates might be reduced.

Based on the above background, our laboratory synthesized a series of CPT-bile acid analogues in order to improve the tumor-targeting chemotherapeutic effects of CPT on liver cancer, thereby decreasing the toxicity of CPT, and evaluated their antitumor activities [22,23]. Among these analogues, the compound **G2** (Figure 1B) showed the highest anticancer activity in vitro and had a broad spectrum of anticancer activity [22]. G2 was synthesized by conjugating the CPT hydroxyl group at C20 position to the deoxycholic acid carboxyl group at C24 position via a linker. In order to evaluate whether **G2** possesses the capacity to target the liver, competitive inhibition experiments were performed to investigate the effects of corresponding bile acid on the anticancer activity and cellular uptake of **G2** in both 2D and 3D HepG2 cell model. Additionally, the in vitro stability of **G2** and CPT was evaluated. Finally, the in vivo distribution of **G2** was also examined in mice, in comparison with CPT.

## 2. Results and Discussion

### 2.1. Effect of Deoxycholic Acid on Cytotoxicity of G2 and CPT

In order to evaluate whether the deoxycholic acid-CPT conjugate **G2** could be taken up by bile acid transporters, a competitive inhibition of antitumor activity experiment was performed. 

**Figure 2:**
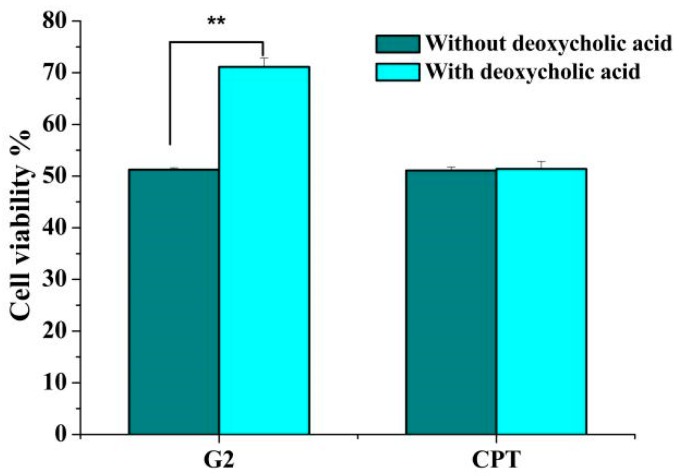
The effect of deoxycholic acid on cytotoxicity of **G2** and CPT. After pretreatment with 1 μM deoxycholic acid for 0.5 h, HepG2 cells were incubated with 1 μM **G2** or CPT for 48 h and the cell viability was detected. Results are presented as mean ± SD of three independent measurements. ** *p* < 0.01 compared with control group.

As shown in Figure 2, pretreatment with deoxycholic acid markedly reduced the cytotoxicity of **G2** against HepG2 cells. After pretreatment with 1 μM deoxycholic acid for 0.5 h, the cell viability of HepG2 exposured to G2 increased from 51.26% to 71.13%. Meanwhile, no distinct effects of deoxycholic acid pretreatment on the cytotoxicity induced by CPT were revealed. Based on these results, we surmised that **G2** could be taken up by bile acid transporters due to its conjugation with deoxycholic acid, which might increase intracellular accumulation of **G2** in HepG2 cells.

### 2.2. Effects of Deoxycholic Acid on Uptake of G2 and CPT in 2D Cell Model

**Figure 3:**
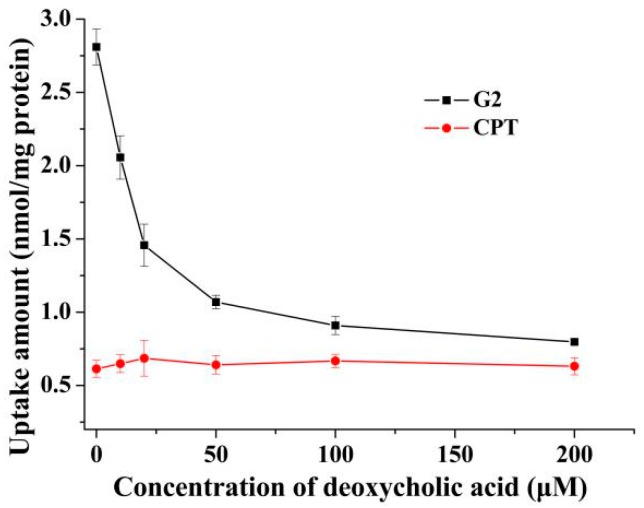
The effect of deoxycholic acid on uptake of **G2** and CPT in HepG2 2D cell model. After pretreatment with different concentrations of deoxycholic acid for 0.5 h, cells were incubated with 10 μM **G2** or CPT for 2 h and the amount of uptake was detected. Results are presented as mean ± SD of three independent measurements.

In order to further confirm the hypothesis, the effects of deoxycholic acid on uptake of **G2** and CPT were investigated. Pretreatment with deoxycholic acid significantly attenuated the uptake of **G2** towards HepG2 cells in 2D cell model in a dose-dependent manner (Figure 3). The uptake of CPT towards HepG2 cells in 2D cell model showed no obvious change after pretreatment with deoxycholic acid. Meanwhile, without deoxycholic acid pretreatment, the uptake of **G2** was 4.16-fold higher than that of CPT. Li et al. also discovered that cholic acid could reduce cellar uptake of cholic acid modified silybin towards HepG2 cells [17]. The inhibitory effect might be exerted via saturating NTCP expressed on HepG2. These results indicated that conjugation with deoxycholic acid changed the transport pathway of CPT and the uptake mediated by bile acid transporters played an important role in the cellular internalization of **G2** in HepG2 cells.

### 2.3. Effects of Deoxycholic Acid on Uptake of G2 and CPT in 3D Cell Model

**Figure 4:**
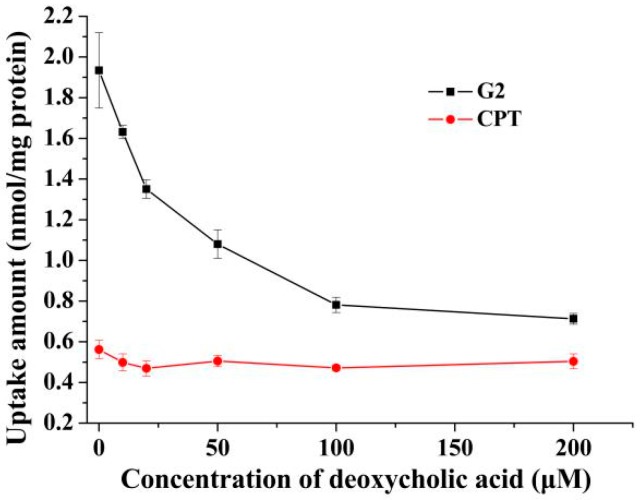
The effect of deoxycholic acid on uptake of G2 and CPT in HepG2 3D cell model. After pretreatment with different concentrations of deoxycholic acid for 0.5 h, spheroids were incubated with 10 μM G2 or CPT for 2 h and the amount of uptake was detected. Results are presented as mean ± SD of three independent measurements.

Although 2D monolayer cell models have been used most frequently for in vitro investigations, they lack the extracellular matrix and 3D architecture of intact tumor tissue [24,25]. Therefore, 2D cell models can’t sufficiently represent the in vivo tumors. In order to further understand the penetration of **G2** and CPT into tumor tissue, a 3D HepG2 spheroid cell model was used to investigate the effect of deoxycholic acid on uptake of **G2** and CPT, for its more similar to intact tumor [26]. As revealed in Figure 4, except for the decreased compound concentration of uptake, similar results to 2D cell model were obtained in 3D cell model. After pretreatment with 200 μM deoxycholic acid, the cellar uptake of **G2** in 3D cell model was decreased 66.4% compared to the group of deoxycholic acid-free. Whereas, there was no distinct effect of deoxycholic acid on cellar uptake of CPT. These results further demonstrated that **G2** might be more exposed in the liver than CPT.

### 2.4. Stability of G2 and CPT

**Figure 5:**
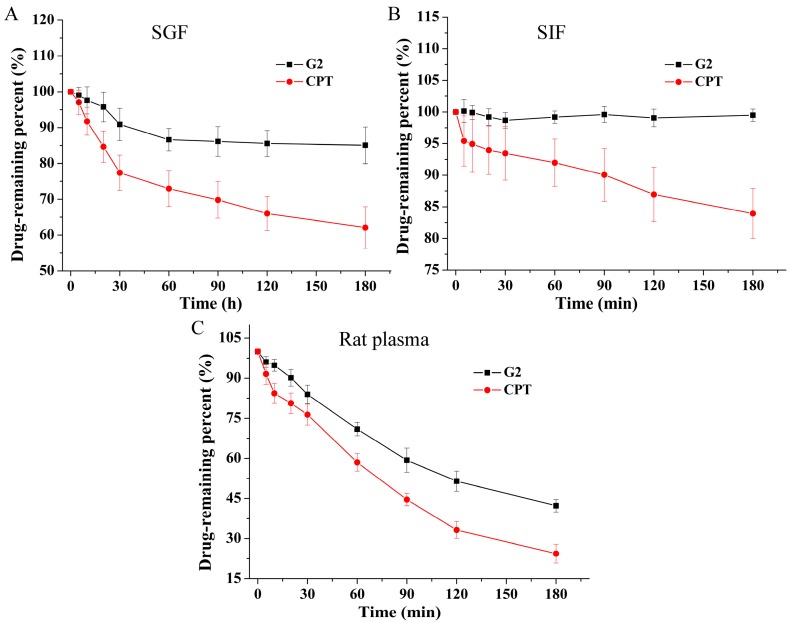
Stability of **G2** and CPT was studied in (**A**) simulated gastric fluid (SGF), (**B**) simulated intestinal fluid (SIF) and (**C**) fresh rat plasma.

Because the instability of the lactone ring is one of the reasons limiting the application of CPT, conjugation with deoxycholic acid at 20-position of CPT was developed to improve its stability and target the liver for its expression of bile acid transporters. Therefore, we measured the stability of **G2** and CPT in simulated gastric fluid (SGF) and simulated intestinal fluid (SIF) as well as fresh rat plasma. As revealed in Figure 5, conjugate **G2** effectively decreased hydrolysis compared with CPT in all of the three media, particularly in SGF and SIF. **G2** was most stable in SIF. After 3 h incubation in SIF, **G2** had no obvious degradation, whereas 16.1% of CPT were degraded. After 3 h incubation in SGF, the degraded rate of **G2** (15.0%) was less than that of CPT (37.9%). 

In rat plasma, **G2** was also more stable than CPT, with 42.3% and 24.4% remaining after 3 h incubation, respectively. These results indicated that conjugation with deoxycholic acid at 20-position of CPT increased the stability of lactone ring, which was consistent with previous reports [27,28]. 

### 2.5. Method Validation

**Figure 6:**
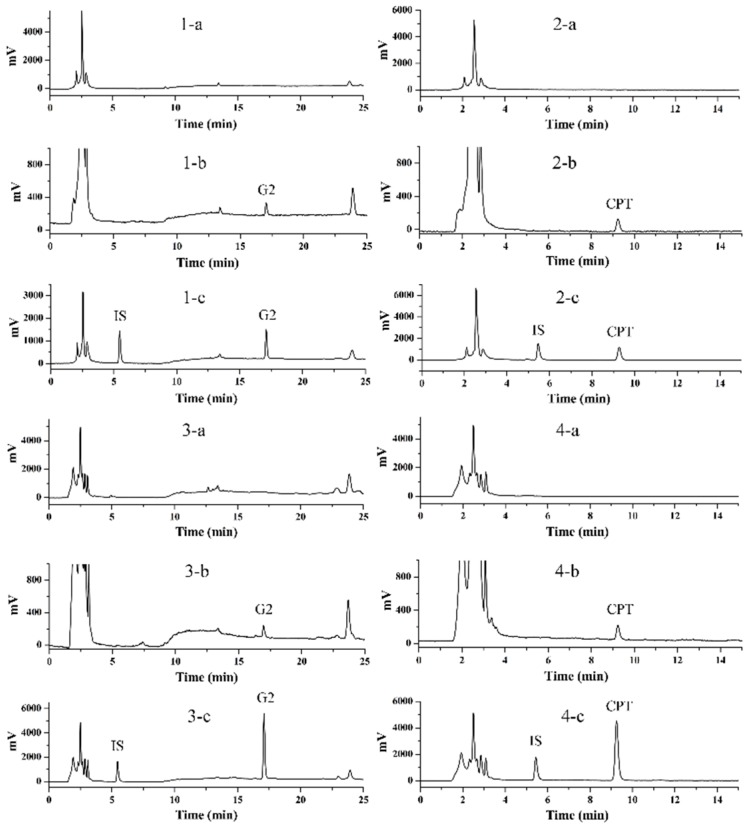
Chromatograms of plasma (1, 2) and liver homogenate (3, 4) samples: (**a**) blank sample; (**b**) blank sample spiked with 0.030 μM **G2** or CPT; (**c**) plasma or liver homogenate sample after oral administration 0.5 h and spiked with IS.

There was no significant endogenous interference for both the plasma and tissue samples (Figure 6). Calibration curves, correlation coefficients and linear ranges were summarized in Table S1 (Supplementary Materials). All correlation coefficients were greater than 0.99, which indicated good linearity. For **G2**, the intra-day precision was no more than 8.90%, and the accuracy was range from 90.67% to 108.24%; the inter-day precision was no more than 6.91%, and the accuracy was range from 93.06% to 104.49% (Table S2, Supplementary Materials). For CPT, the intra-day precision was no more than 8.11%, and the accuracy was range from 95.22% to 105.54%; the inter-day precision was no more than 6.08%, and the accuracy was range from 94.73% to 106.18% (Table S2). Recovery in plasma and tissue homogenates were ranged from 82.40% to 91.48% and 81.66% to 93.06% for **G2** and CPT, respectively (Table S3, Supplementary Materials). Furthermore, there was no significant matrix effect for the determination of **G2** and CPT (Table S3). For the freeze-thaw, long-term and short-term stability investigations, the concentrations of **G2** and CPT in plasma and tissue homogenates diverged no more than ±15% from nominal values (Table S4, Supplementary Materials). 

### 2.6. Body Distribution

**Figure 7:**
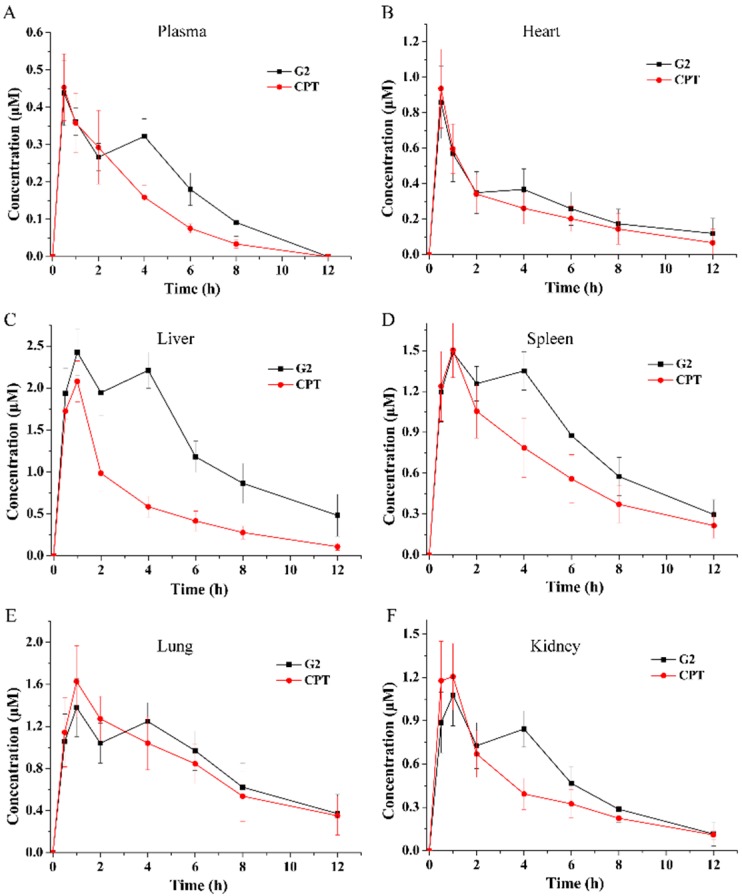
Total drug concentration-time profiles of **G2** and CPT in plasma and organs after oral (30 mg/kg, calculated as CPT) administration: (**A**) plasma; (**B**) heart; (**C**) liver; (**D**) spleen; (**E**) lung; (**F**) kidney. Results are presented as mean ± SD of five mice.

Based on the results of the in vitro studies, a biodistribution of **G2** and CPT was investigated in mice to evaluate the potential of liver targeting of deoxycholic acid conjugate **G2** in vivo. After **G2** or CPT intragastric administration, the concentrations of **G2** or CPT were determined in the plasma and organs. The mean concentration-time curves of **G2** and CPT in plasma and tissues were showed in Figure 7 and the main pharmacokinetic parameters obtained utilizing non-compartmental model were presented in Table 1. For CPT, the AUC_0–t_ was highest in lung, but it was highest in liver for G2. This result indicated that conjugation with deoxycholic acid increased liver accumulation. In addition, compared with CPT, the AUC of deoxycholic acid conjugate **G2** increased in all of the plasma and organs. Meanwhile, it increased most significantly in liver than other organs. After oral administration, the AUC_0–t_ of **G2** in liver was 16.04 ± 2.78 nmol h/mL, which was 2.31-fold higher than that of CPT. And the LTI was 8.07 and 4.84 for **G2** and CPT, respectively. It demonstrated that conjugation with deoxycholic acid increased liver targeting in vivo by 1.67-fold in comparison to CPT. This result was in accordance with previous report that conjugation with bile acid could increase liver targeting of ribavirin by 1.80-fold [16].

## 3. Materials and Methods

### 3.1. Materials 

The deoxycholic acid-CPT conjugate **G2** (Figure 1B, CPT-20 (S)-*O*-acetate-deoxycholic acid) was synthesized and purified by our laboratory [22], to a purity beyond 99%. The human hepatocellular carcinoma cell line HepG2 was purchased from the China Center for Type Culture Collection (Wuhan, China). Dulbecco’s modified Eagle’s medium (DMEM) was obtained from Gibco BRL (Grand Island, NY, USA). Dimethyl sulfoxide (DMSO) was purchased from Sigma-Aldrich (St. Louis, MO, USA). Fetal bovine serum (FBS) was obtained from Hyclone (Thermo Fisher Scientific, Logan, UT, USA). Phosphate-buffered saline (PBS), Hank’s buffered salt solution (HBSS), penicillin and streptomycin were supplied by Genom (Hangzhou, China). Methyl thiazolyl tetrazolium (MTT) and trypsin were obtained from Solarbio (Beijing Solarbio Science & Technology Co., Ltd., Beijing, China). All reagents for HPLC were of analytical grade.

### 3.2. Cell Culture

The human hepatocellular carcinoma cell line HepG2 were cultured in T-25 cm^2^ cell culture flasks (Corning^®^, Wujiang, China) with media consisting of DMEM supplemented with 10% (*v/v*) FBS, 100 U/mL penicillin, and 100 μg/mL streptomycin. Cells were maintained at 37 °C with 5% CO_2_ and 95% relative humidity. After 90% confluence, cells were digested by trypsin and passaged at a 1:3 split ratio.

### 3.3. 3D Multicellular Tumor Spheroids Culture

The liquid overlay method was employed for the culturing of HepG2 spheroids as described previously [29]. Cells reached logarithmic growth phase were digested by trypsin then centrifuged. The cell number was counted using a hemocytomete and cells were diluted in growth medium to a density of 1 × 10^4^ cells/mL. Spheroids were seeded by pipetting 2 mL of the cell suspension into each well of a 6-well ultra-low attachment (ULA) plates (Corning^®^, Wujiang, China) and incubated for 3 days. 

### 3.4. Competitive Inhibition Assays 

The effect of deoxycholic acid on anticancer activity of **G2** against HepG2 cells was studied by MTT assay as described previously with slight modifications [30]. After reaching logarithmic growth phase, HepG2 cells were passaged and 100 μL of the cell suspension was seeded into 96-well plates at a density of 5 × 10^4^ cells/mL, then cells were cultured in an incubator (Thermo Fisher Scientific, Marietta, Ohio, USA) at 37 °C. After reached 80% confluence, cell monolayers were firstly incubated with or without 1 μM deoxycholic acid for 0.5 h. After pretreatment with deoxycholic acid, cells were washed with PBS buffer and 200 μL of 1 μM CPT or **G2** was added. Next, cells were further incubated at 37 °C for 48 h. Then 20 μL of MTT reagent (5 mg/mL in PBS) was added and cells were incubated for another 4 h at 37 °C to allow MTT to be metabolized into formazan. Finally, the media was tapped out and 150 μL of DMSO was added to each well to dissolve the formazan crystals. After mixing, the absorbance was quantified at 490 nm using an ELISA Plate Reader (infinite M200 Pro, TECAN, Grodig, Salzburg, Austria).

### 3.5. Uptake of G2 into 2D Cell Model 

Cellular uptake of **G2** and CPT into 2D HepG2 cell model was performed as described previously with slight modifications [31]. After reaching logarithmic growth phase, HepG2 cells were passaged and 2 mL of the cell suspension was seeded into 6-well plates at a density of 5 × 10^4^ cells/mL. Then, cells were cultured in an incubator at 37 °C. After reached 80% confluence, cell monolayers were pretreated with deoxycholic acid at different concentrations for 0.5 h. Next, the inhibitor of deoxycholic acid was removed and cells were washed twice with Hank’s balanced salt solution (HBSS). After 2 h incubation with 10 μM **G2** or CPT, cells were washed three times with HBSS and digested with 200 μL of trypsin for 2 min to obtain single cell suspensions. Next, the cells were washed three times with cold HBSS and 250 μL of HBSS was added to resuspend the pellets. Then, the cells were lysed by freeze-thaw three times and sonicated for 10 min. After centrifugation, the supernatants were used for the measure of the compound and protein concentration. For determination of the compound concentration, 200 μL of the supernatant was added to equal volume of acetonitrile to precipitate proteins, then the mixture was vortexed and mixed for 2 min. After centrifugation, the supernatant was collected and filtered with a 0.22 μm filter to acquire the sample for HPLC analysis. The compound concentration was calculated by the corresponding standard curve. The protein concentration of the supernatant was determined by the Bradford method based on a standard curve made by desired concentrations of bovine serum albumin. The cellular uptake was presented as the quantity of **G2** or CPT associated with 1 mg of cellular protein [32,33].

### 3.6. Uptake of G2 into 3D Cell Model

After HepG2 spheroids were prepared as 3.3, they were transferred into 4 mL centrifuge tube and washed twice with HBSS. Then, the spheroids were resuspended with deoxycholic acid at different concentrations and seeded into the 6-well ULA plates again. After being cultured in an incubator for 0.5 h at 37 °C, the spheroids were transferred into 4 mL centrifuge tube and washed twice with HBSS to remove the deoxycholic acid. Then, they were resuspended with 10 μM **G2** or CPT and seeded into the 6-well ULA plates. After being cultured in an incubator for 2 h at 37 °C, the spheroids were transferred into 4 mL centrifuge tube and washed three times with HBSS. Then, the spheroids were digested with 200 μL of trypsin for 5 min to obtain single cell suspensions and subsequent processing was the same as 2D cell model.

### 3.7. Stability of G2 

The stability of **G2** and CPT was assessed in SGF, SIF and fresh rat plasma. SGF and SIF were prepared according to a previous report with slight modifications [34]. Briefly, 0.3% (*w/v*) purified pepsin, 2 g of NaCl and 7 mL of HCl (12 M) were dissolved in 1000 mL of distilled water to obtain SGF (PH 1.2). To make SIF (PH 6.8), 6.8 g of KH_2_PO_4_, 1.0% (*w/v*) purified pancreatin and 77 mL of NaOH (0.2 M) were dissolved in 1000 mL of distilled water. SGF, SIF and rat plasma containing 10 μM **G2** or CPT were incubated at 37 °C. At scheduled time points (0, 5, 10, 20, 30, 60, 90, 120, 180 min), 100 μL samples were collected and reaction was quenched immediately by adding 400 μL of ice cold acetonitrile. The mixture was votexed 2 min and centrifuged at 12000 rpm for 10 min. Then, the supernatant was filtered with a filter (0.22 μm) and detected by HPLC. 

### 3.8. In Vivo Distribution of G2 in Mice

For body distribution investigations, 80 male Kunming mice (weight, 18–22 g; age, 6–8 weeks) were used. The animal experiments in this study were performed under the guidance of the care and use of laboratory animals in Zhejiang University of Technology, Hangzhou, China, and conformed to the National Institutes of Health Guide for Care and Use of Laboratory Animals (Publication No. 85-23). After fasting overnight, mice were received a single dose of **G2** or CPT (30 mg/kg, calculated as CPT) suspension by gavage. At desired time (0.5, 1, 2, 4, 6, 8, 12 h), animals were sacrificed. Their blood samples were collected by eyeball removal and immediately centrifuged to separate plasma. The organs (heart, liver, spleen, lung, kidney) were removed and weighed before homogenization. And the plasma and tissue homogenate samples were stored at −80 °C until analysis.

### 3.9. Calibration Curves and Quality Control Samples Preparation

**G2**, CPT and 10-hydroxycamptothecin (IS) were dissolved in DMSO at 1 mM to obtain the standard stock solutions. The standard working solutions (0.3, 0.6, 1.2, 3, 6, 12, 24, 48 μM for **G2** and CPT; 2 μM for IS) were prepared by diluting the stock solutions with acetonitrile. The calibration standards (0.015, 0.03, 0.06, 0.15, 0.3, 0.6, 1.2, 2.4 μM) were prepared by spiking 5 μL of standard working solutions into 95 μL of blank plasma or tissue homogenate. The preparation of quality control samples (0.03, 0.3, 1.2 μM) was same as the calibration standards. All solutions were stored at 4 °C until further use.

### 3.10. Plasma and Tissue Homogenate Samples Processing

For determination of the compound concentration, 200 μL methanol and 100 μL acetonitrile were added to 100 μL plasma or tissue homogenate to precipitate protein, and 5 μL IS (2 μM) was also added. Mixtures were votexed for 2 min and centrifuged at 12,000 rpm for 10 min at 4 °C. Then, supernatant was transferred to a new tube and evaporated by an EZ-2 personal solvent evaporator (Genevac, Suffolk, UK). Finally, 50 μL of 50% acetonitrile (*v/v*) was added to re-dissolve the samples. After centrifuged at 13,000 rpm for 10 min, the supernatant was used for HPLC analysis.

### 3.11. HPLC Analysis

Both **G2** and CPT were measured by HPLC analysis, which was performed using a Shimadzu HPLC system (Shimadzu, Kyoto, Japan). To determine **G2** and CPT concentration in both cell models, an Inertsil ODS-SP C18 column (5 μm, 4.6 × 250 mm; GL Sciences, Tokyo, Japan) was used. The column was maintained at 30 °C and the absorbance detector wavelength was 363 nm. The chromatographic separation was performed on isocratic elution with acetonitrile-0.1% formic acid (80:20, *v/v*) and acetonitrile-0.1% formic acid (45:55, *v/v*) for **G2** and CPT, respectively. The flow velocity was 1.0 mL/min. The injection volume of the supernatant samples was 20 μL.

To determine the concentration of **G2** and CPT in the stability investigation, a C18 (5 μm, 4.6 × 250 mm, Elite, Dalian, China) reverse-phase column was used. The chromatographic separation was performed on isocratic elution with acetonitrile-0.1% formic acid (80:20, *v/v*) and acetonitrile-0.1% formic acid (60:40, *v/v*) for **G2** and CPT, respectively. Other conditions were the same as cell model.

To determine **G2** and CPT concentration in plasma and tissue homogenate, an Inertsil ODS-SP C18 column (5 μm, 4.6 × 250 mm; GL Sciences) was used. The mobile phase was made up of acetonitrile (A) and 0.1% formic acid (B). CPT was separated by an isocratic elution with 35:65 (*v/v*) A:B. A gradient eluant ((A:B) 0–3 min: 35:65; 3–8 min: from 35:65 to 80:20; 8–20 min: 80:20; 20–21 min: from 80:20 to 35:65; 21–25 min: 35:65) was used to separate **G2**. Other conditions were the same as cell model.

### 3.12. Method Validation

As **G2** and CPT were studied in vivo, the analytical method was validated. Method validation was conducted according to the acceptance criteria established by U.S. Food and Drug Administration for bioanalytical method validation. The validation parameters included specificity, linearity, accuracy, precision, recovery, matrix effect and stability.

The selectivity of the method was assessed by comparing the chromatograms of six different sources of blank samples with the corresponding spiked samples. The calibration curves were constructed by plotting the peak area ratio of **G2** or CPT to IS (y) versus the concentration (x, range from 0.015 to 2.4 μM). The regression equations were obtained using least squares linear regression weighted by 1/x^2^. The inter-day and intra-day accuracy and precision were evaluated using three concentrations (0.03, 0.3, 1.2 μM) of QC samples on the same day and different validation days, respectively. The accuracy was represented by the percentage (%) of mean measured concentration to spiked concentration. Precision was represented by relative standard deviation (RSD). Extraction recovery was calculated by dividing the peak areas of QC samples at three concentrations with the unextracted standards. The stability was evaluated using QC samples at three concentrations to confirm freeze-thaw stability (three cycles), long-term freeze stability (− 80 °C for four weeks), short-term stability (room temperature for 4–24 h). The results of stability were expressed as the percentage (%) of mean measured concentration in this experiment to the initial concentration. The matrix effect was obtained via comparing the peak areas of spiked solution of post-extracted blank plasma with pure standard solution.

### 3.13. Data Analysis

The pharmacokinetic analysis using a non-compartmental model with the PKSolver pharmacokinetic software (China Pharmaceutical University, Nanjing, China) was conducted to calculate the key parameters of **G2** and CPT. In order to evaluate whether the conjugate had the trend of liver targeting, liver targeting index (LTI) was calculated by Equation (1) below:

LTI = AUC_0–t’_ in liver/AUC_0–t_ in plasma (1)

where AUC_0–t’_ in liver is the AUC_0–t_ of drug in liver and AUC_0–t_ in plasma is the AUC_0–t_ of drug in plasma. All the data were presented as mean ± SD. Statistical analysis were performed by Student’s *t*-test using the SPSS 22.0 statistical software program (IBM company, Denver, CO, USA), and *p* < 0.05 was used as the criterion for statistical significant.

## 4. Conclusions

In this interesting study, we evaluated the potential liver targeting and stability of deoxycholic acid-CPT conjugate **G2** in vitro as well as its potential liver targeting in vivo. The results of competitive inhibition of antitumor activitiy against HepG2 cells suggested that **G2** could be taken up by bile acid transporters for its conjugation with deoxycholic acid. Subsequent investigation of uptake into HepG2 cells in 2D cell model and 3D model further demonstrated that conjugation with deoxycholic acid changed the transport pathway of CPT and the uptake mediated by bile acid transporters played an important role in the cellular internalization of **G2** in HepG2 cells. Stability tests in SGF and SIF as well as rat plasma showed that conjugation with deoxycholic acid significantly increased the stability in vitro. Body distribution study in mice demonstrated that **G2** increased the LTI by 1.67-fold compared with CPT. In short, the current study indicates that coupling with deoxycholic acid moiety is a feasible means to enhance the potential liver targeting and the stability of CPT, which provide a theoretical basis for preclinical trials of G2.

## Figures and Tables

**Table 1:** Pharmacokinetic parameters of **G2** and CPT after oral (30 mg/kg, calculated as CPT) administration (mean ± SD, n = 5).

Analyte	Organ	T_max_ (h)	C_max_ (nmol/mL)	AUC_0–t_ (nmol h/mL)	AUC_0–∞_ (nmol h/mL)	MRT_0–∞_ (h)
**G2**	Plasma	0.600 ± 0.200	0.441 ± 0.084	1.987 ± 0.319	2.303 ± 0.500	4.355 ± 0.645
	Heart	0.600 ± 0.200	0.899 ± 0.165	3.362 ± 1.065	4.589 ± 1.937	7.799 ± 3.102
	Liver	1.000 ± 0.000	2.425 ± 0.279	16.043 ± 2.783	20.093 ± 6.046	7.194 ± 2.298
	Spleen	1.600 ± 1.200	1.526 ± 0.140	10.371 ± 1.382	12.112 ± 2.056	6.379 ± 1.352
	Lung	1.600 ± 1.200	1.386 ± 0.269	10.180 ± 2.318	12.889 ± 4.211	7.446 ± 1.748
	Kidney	1.800 ± 1.166	1.096 ± 0.193	6.055 ± 1.197	6.627 ± 1.740	5.049 ± 1.003
CPT	Plasma	0.500 ± 0.000	0.465 ± 0.084	1.556 ± 0.260	1.677 ± 0.252	3.224 ± 0.578
	Heart	0.600 ± 0.200	0.954 ± 0.207	2.860 ± 0.983	3.639 ± 1.615	6.350 ± 2.712
	Liver	1.000 ± 0.000	2.079 ± 0.243	6.954 ± 1.319	7.459 ± 1.539	4.261 ± 0.380
	Spleen	0.900 ± 0.200	1.526 ± 0.195	7.562 ± 1.718	9.292 ± 2.527	6.958 ± 2.948
	Lung	1.200 ± 0.400	1.641 ± 0.325	9.798 ± 2.594	12.431 ± 4.429	7.248 ± 1.775
	Kidney	0.800 ± 0.245	1.328 ± 0.218	4.827 ± 1.045	5.448 ± 1.193	5.322 ± 0.460

## Supplementary Materials

Tables S1–S4 are available on line. Table S1: Linearity of G2 and CPT in biological samples (n = 3). Table S2: Accuracy and precision data for G2 and CPT in biological samples (n = 6). Table S3: Recovery and matrix effect of G2 and CPT in biological samples (n = 6). Table S4: Stability data for G2 and CPT in biological samples (n = 6).
