# Supplementary material for: Enhanced Liver Targeting of Camptothecin via Conjugation with Deoxycholic Acid
Source: Molecules. 2019 Mar 26;24(6):1179. doi: 10.3390/molecules24061179 (PMC6472190; doi:10.3390/molecules24061179)
Supplement: Supplementary file 1 [file molecules-24-01179-s001.pdf]

Enhanced Liver Targeting of Camptothecin via Conjugation with Deoxycholic Acid

Linxia Xiao [1], Endian Yu [2], Hanlin Yue [2] and Qingyong Li [1,2,*]

[1] Collaborative Innovation Center of Yangtze River Region Green Pharmaceuticals, Zhejiang University of Technology, Hangzhou 310014, China; xiaolinxiaxlx@126.com

[2] College of Pharmaceutical Science, Zhejiang University of Technology, Hangzhou 310014, China; yu19960216@126.com (E.Y.); yhl333666@126.com (H.Y.)

* Correspondence: liqy@zjut.edu.cn; Tel.: +86-571-88320984

Table S1. Linearity of G2 and CPT in biological samples (n = 3).

Analyte	Biological samples	Calibration curves	Correlation coefficients (r)	Liner range (μM)
G2	Plasma	Y = 1.7535 x + 0.0014	0.9934	0.015-2.4
	Heart	Y = 1.7312 x + 0.0005	0.9944	0.015-2.4
	Liver	Y = 1.5794 x + 0.0030	0.9927	0.015-2.4
	Spleen	Y = 1.5919 x − 0.0001	0.9939	0.015-2.4
	Lung	Y = 1.6238 x + 0.0024	0.9943	0.015-2.4
	Kidney	Y = 1.7999 x + 0.0002	0.9919	0.015-2.4
CPT	Plasma	Y = 1.8646 x + 0.0015	0.9963	0.015-2.4
	Heart	Y = 1.8024 x + 0.0003	0.9919	0.015-2.4
	Liver	Y = 1.8105 x + 0.0022	0.9932	0.015-2.4
	Spleen	Y = 1.7392 x + 0.0039	0.9968	0.015-2.4
	Lung	Y = 1.7984 x + 0.0015	0.9988	0.015-2.4
	Kidney	Y = 1.7959 x + 0.0043	0.9911	0.015-2.4

Table S2. Accuracy and precision data for G2 and CPT in biological samples (n = 6).

Analyte	Biological samples	Nominal concentration (μM)	Intra-day		Inter-day	
			Accuracy (mean, %)	Precision (RSD, %)	Accuracy (mean, %)	Precision (RSD, %)
G2	Plasma	0.03	106.26	8.24	98.70	6.91
		0.3	101.12	3.20	97.80	4.60
		1.2	99.29	3.28	96.67	2.49
	Heart	0.03	91.82	7.59	96.69	6.19
		0.3	95.81	8.03	93.06	4.63
		1.2	97.82	4.36	97.21	2.07
	Liver	0.03	96.25	5.43	97.42	4.94
		0.3	93.79	4.77	97.20	4.30
		1.2	98.26	2.72	99.67	1.41
	Spleen	0.03	108.24	6.21	104.49	3.80
		0.3	104.25	7.43	99.59	4.10
		1.2	104.98	6.92	101.63	3.04
	Lung	0.03	90.67	7.31	93.26	5.14
		0.3	92.37	5.39	95.08	2.55
		1.2	98.38	2.09	96.99	1.59
	Kidney	0.03	93.30	7.03	94.35	5.29
		0.3	93.34	8.90	93.58	5.08
		1.2	97.95	2.21	96.73	3.67
CPT	Plasma	0.03	101.90	6.79	101.07	4.57
		0.3	97.99	6.90	98.93	5.20
		1.2	98.27	2.52	94.73	3.40
	Heart	0.03	102.13	8.11	105.75	3.34
		0.3	104.05	5.54	104.13	5.40
		1.2	98.96	1.19	103.69	4.02
	Liver	0.03	105.54	5.78	102.27	3.22
		0.3	104.39	4.06	103.02	4.15
		1.2	101.03	2.15	98.99	1.85
	Spleen	0.03	95.22	5.72	101.95	6.08

		0.3	99.11	4.43	106.18	5.84
		1.2	100.13	3.90	104.15	3.36
	Lung	0.03	97.51	4.38	103.64	5.15
		0.3	103.82	5.11	102.62	4.12
		1.2	100.94	1.91	96.61	4.71
	Kidney	0.03	97.36	7.22	99.03	5.08
		0.3	103.56	4.76	102.98	3.93
		1.2	99.23	2.15	99.91	5.15

Table S3. Recovery and matrix effect of G2 and CPT in biological samples (n = 6).

Analyte	Biological samples	Nominal concentration (μM)	Recovery (mean ± SD, %)	Matrix effect (mean ± SD, %)
G2	Plasma	0.03	83.13 ± 2.82	99.79 ± 3.39
		0.3	87.44 ± 3.28	99.55 ± 2.76
		1.2	91.48 ± 5.39	98.04 ± 5.88
	Heart	0.03	82.40 ± 6.54	102.51 ± 3.93
		0.3	85.10 ± 3.23	99.44 ± 3.75
		1.2	87.66 ± 3.83	102.84 ± 3.13
	Liver	0.03	84.27 ± 5.47	99.83 ± 3.98
		0.3	87.99 ± 2.73	94.60 ± 3.75
		1.2	89.97 ± 1.73	93.53 ± 1.33
	Spleen	0.03	82.45 ± 7.70	103.13 ± 4.45
		0.3	89.34 ± 3.79	95.75 ± 5.48
		1.2	87.25 ± 4.68	102.73 ± 3.04
	Lung	0.03	89.04 ± 3.71	97.37 ± 3.81
		0.3	88.13 ± 5.28	98.19 ± 3.99
		1.2	88.69 ± 2.42	97.43 ± 2.13
	Kidney	0.03	88.49 ± 8.12	94.97 ± 4.08
		0.3	86.19 ± 6.44	105.16 ± 3.71
		1.2	85.04 ± 3.95	105.34 ± 2.77
CPT	Plasma	0.03	84.30 ± 4.40	97.64 ± 2.56
		0.3	86.19 ± 4.67	99.58 ± 3.41
		1.2	84.96 ± 3.66	101.76 ± 3.07
	Heart	0.03	82.52 ± 7.70	102.83 ± 4.19
		0.3	88.40 ± 4.47	93.62 ± 2.70
		1.2	88.24 ± 4.63	103.74 ± 2.49
	Liver	0.03	87.99 ± 5.31	97.49 ± 3.50
		0.3	84.05 ± 3.82	102.01 ± 3.17
		1.2	84.12 ± 2.50	103.67 ± 1.89
	Spleen	0.03	83.69 ± 5.68	100.14 ± 2.72
		0.3	89.40 ± 4.38	94.90 ± 3.04
		1.2	93.06 ± 4.44	95.97 ± 2.27
	Lung	0.03	85.17 ± 2.60	99.92 ± 1.89
		0.3	86.33 ± 4.25	95.94 ± 2.65
		1.2	90.21 ± 3.46	95.52 ± 2.06
	Kidney	0.03	85.25 ± 6.44	101.20 ± 3.86
		0.3	81.66 ± 4.54	103.45 ± 4.33
		1.2	89.85 ± 5.56	100.70 ± 3.38

Table S4. Stability data for G2 and CPT in biological samples (n = 6).

Analyte	Biological samples	Nominal concentration (μM)	Free-thaw stability accuracy (mean ± SD, %)	Short-term stability accuracy (mean ± SD, %)	Long-term stability accuracy (mean ± SD, %)
G2	Plasma	0.03	92.83 ± 6.37	93.83 ± 6.69	95.44 ± 3.14
		0.3	97.42 ± 6.36	98.29 ± 6.12	95.57 ± 3.50
		1.2	97.68 ± 3.99	97.48 ± 1.75	103.39 ± 4.75
	Heart	0.03	102.78 ± 5.28	101.06 ± 7.39	102.44 ± 6.05
		0.3	98.26 ± 5.50	94.57 ± 6.38	99.39 ± 7.00
		1.2	99.95 ± 3.39	96.89 ± 2.88	95.57 ± 2.20
	Liver	0.03	97.94 ± 7.28	97.17 ± 4.02	96.33 ± 7.01
		0.3	98.56 ± 6.82	99.41 ± 2.97	97.39 ± 6.66
		1.2	100.14 ± 2.13	100.86 ± 2.08	99.60 ± 2.74
	Spleen	0.03	96.67 ± 5.42	95.50 ± 7.03	100.56 ± 7.48
		0.3	98.23 ± 7.95	103.52 ± 6.27	96.71 ± 5.01
		1.2	98.08 ± 1.33	98.45 ± 3.48	99.45 ± 3.92
	Lung	0.03	95.06 ± 8.42	102.22 ± 4.55	98.06 ± 5.77
		0.3	99.37 ± 5.64	98.20 ± 4.69	101.95 ±5.16
		1.2	98.66 ± 2.45	97.98 ± 2.60	97.59 ± 3.16
	Kidney	0.03	97.89 ± 7.09	94.22 ± 7.59	92.11 ± 4.70
		0.3	102.45 ± 7.46	95.03 ± 6.62	98.18 ± 5.29

		1.2	99.79 ± 2.80	97.89 ± 1.86	101.49 ± 6.35
CPT	Plasma	0.03	99.06 ± 6.03	98.61 ± 7.53	98.22 ± 6.93
		0.3	103.39 ± 7.35	102.22 ± 3.71	102.31 ± 6.63
		1.2	98.62 ± 1.61	97.86 ± 2.44	98.54 ± 4.17
	Heart	0.03	98.27 ± 8.16	96.11 ± 6.61	93.67 ± 4.98
		0.3	96.26 ± 5.80	97.33 ± 5.72	96.07 ± 6.34
		1.2	99.23 ± 5.06	100.22 ± 2.58	102.74 ± 4.45
	Liver	0.03	101.39 ± 6.16	96.83 ± 5.43	94.06 ± 4.92
		0.3	98.08 ± 8.17	102.33 ± 4.10	98.59 ± 5.15
		1.2	98.88 ± 3.46	98.93 ± 2.93	97.12 ± 2.67
	Spleen	0.03	94.44 ± 6.60	97.78 ± 4.87	97.22 ± 7.80
		0.3	99.13 ± 8.21	95.13 ± 4.94	97.17 ± 5.32
		1.2	103.34 ± 4.90	98.91 ± 3.18	99.76 ± 1.94
	Lung	0.03	97.56 ± 7.89	93.22 ± 5.68	103.72 ± 5.32
		0.3	98.76 ± 7.57	98.14 ± 4.96	105.09 ± 3.90
		1.2	97.82 ± 4.50	97.83 ± 1.76	96.18 ± 3.39
	Kidney	0.03	96.39 ± 7.83	92.39 ± 5.89	101.83 ± 7.43
		0.3	101.84 ± 6.53	94.34 ± 6.64	98.51 ± 6.72
		1.2	97.71 ± 2.41	97.40 ± 2.90	96.65 ± 3.84
